# In Vitro and in Vivo Scolicidal Activities of *Holothuria leucospilota* Extract and CeO2 Nanoparticles against Hydatid Cyst

**Published:** 2019

**Authors:** Safa ARYAMAND, Shahram KHADEMVATAN, Khosrow HAZRATI TAPPEH, Behnam HESHMATIAN, Ali JELODAR

**Affiliations:** 1. Cellular and Molecular Research Center, Department of Medical Parasitology and Mycology, Urmia University of Medical Sciences, Urmia, Iran; 2. Department of Physiology, Urmia University of Medical Sciences, Urmia, Iran; 3. Department of Medical Parasitology, Jondishapur University of Medical Sciences, Ahvaz, Iran

**Keywords:** Hydatid cyst, *Holothuria leucospilota*, CeO2 NPs, CASPASE-3 activity

## Abstract

**Background:**

We aimed to investigate the scolicidal effects of *Holothuria leucospilota* extract and CeO2 nanoparticles against protoscoleces of hydatid cysts in-vitro and in-vivo.

**Methods:**

Hydatid cysts were collected from, Urmia slaughterhouses between years 2016–2017 and the hydatid fluid aspirated from the fertile cysts. Various concentration of *H. leucospilota* extract, CeO2 NPs and combination of CeO2-NPs/*H. leucospilota* were used for 10–60 min to evaluate the viability of protoscoleces by 0.1% eosin method. CASPASE -3 activity measured for assessment of cell apoptosis in treated protoscoleces. BALB/c mice were infected intraperitoneally with 2000 viable protoscoleces and treated daily for 4 wk by intragastrical inoculation with *H. leucospilota*, CeO2 NPs, combination of CeO2 NPs/*H. leucospilota* and Albendazole. Cyst development was macroscopically analyzed.

**Results:**

*H. leucospilota* extract and combination of CeO2 NPs/*H. leucospilota* have potent scolicidal activity at concentration of 20 mg/ml and 15 mg/ml after 60 min treatment. Maximum caspase-3 activity was observed when protoscoleces expose with *H. leucospilota* and combination *H. leucospilota* & CeO2 NPs. After treatment of cyst infected mice with extract and CeO2 NPs, combination of CeO2 NPs/*H leucospilota* and albendazole, a significant decrease in number of cysts, size and volume of cyst (*P*<0.05) was observed.

**Conclusion:**

This result shows an antihydatic and scolicidal effects of *H. leucospilota* extract and CeO2 *NPs*.

## Introduction

Cystic echinococcosis (CE) or hydatidosis is a chronic zoonotic parasitic infection caused by *Echinococcus granulosus*. The life cycle of *E. granulosus* involves dogs and wild carnivores as a definitive host for the adult tapeworm and sheep, goats, cattle and camels as an intermediate host for the larval stage. Human can also be an accidental intermediate host for *E. granulosus* and infected through ingestion of parasite eggs in contaminated food, water or soil, or through direct contact with animal hosts (1).

Echinococcosis has a worldwide spread and considerable public health and economical concerns in many countries of world (1–3). Prevalence of disease was estimated at about 1–220/100000 in world. Hydatidosis in Iran is one of the most common zoonoses infection in endemic areas especially in rural and nomadic areas with sheep husbandry jobs (4–6). Human echinococcosis is reported from many parts of Iran. About 1% of patients referred to surgical wards, patients with hydatid cyst and prevalence of human hydatidosis is (1.2–0.6)/100000 in Iran (1, 4–6).

Hydatid cyst affects different organs. The most common organ is the liver (50%–70%) and lungs (20%–30%) and less frequently in the bones, kidneys, spleen, muscles, central nervous system and eyes (7,8). Thus, rapid diagnosis of disease and the use of effective therapies, before and after surgery, can be effective in controlling and treatment of disease (7,9).

Surgery is still main treatment of CE, however there is increasing risks of frequent recurrences, cyst rupture and spillage of protoscoleces fluid and secondary infections at the same organ or other organs (7, 8). Therefore, use of suitable drug therapy, before and after surgery and during aspirations of cysts, can be effective to reduce recurrence, secondary hydatidosis, further surgery and can completely cure the diseases (9). Now according to recommendations of WHO, albendazole and/or mebendazole used in treatment of CE but these synthetic drugs have various side effects, resistance to live, and active pathogens (10, 11).

To be more effective of drug therapies, the specified concentrations of the drugs must be able to pass through layers of cysts and remove the live protoscoleces of hydatid cyst (12). Thus, the use of new scolicidal drugs with high efficacy in a shorter time of exposure, fewer side effects, less toxicity and less drug resistance, is an essential need for surgeons (13). One of the helpful agents in treatment of hydatidosis is use of nanoparticles (14).

In recent years considerable attention to nanoparticles and used in treatment. Controlled delivery of the drug to the target organs reduce side effects, low toxicity, increase drugs absorptions and increase the effectiveness of treatment of diseases are the advantages of nanoparticles (15). Cerium oxide (CeO2) is one of the effective therapeutic nanoparticles with important rules in human health. So far, it has been used in different medical therapies and studies such as antioxidant effects, antiplasmodium reduction potential, wound healing, antibacterial and reducing nematode longevity (16, 17).

One of the other therapies use of bioactive compounds extracts from sponge, coral, crustacean, fish and echinoderm (18). Sea cucumbers (*Holothuria leucospilota*) are echinoderms from the class Holothuroidea located in the deep seas all over the world. Recently they have been the attention of many researchers. The various extracts derived from sea cucumbers have many activities such as antiviral, antibacterial, antiparasitic (19), anticancer, antioxidant, antifungal and anti-inflammatory (20–22). The presence of compounds such as saponin, chondroitin sulfate, Glucosamine, glycosphingolipid and essential fatty acid is the origin of many biological properties in these animals. Based on existing research, sea cucumber extract can inhibit tumor activity through induction of apoptosis (23, 24).

Apoptosis is a process of programmed cell death, that in normal conditions would remove old, excess and harmful cells and it is essential for homeostasis. Apoptosis can be initiated through one of two pathways, intrinsic pathway (mitochondrial) and extrinsic pathway (death ligand) (25). Both pathways induce cell death by activating caspases, which are proteases, or enzymes that have basic rules in the initiation and execution of apoptosis. As an executioner caspase, the caspase-3 plays a central role in the execution-phase of cell apoptosis (26).

We investigated the scolicidal effects of *H. leucospilota* extract and CeO2 nanoparticles against protoscolices of hydatid cysts on in-vitro and in-vivo model.

## Methods

### Chemicals

CeO2-NPs (Cerium Oxide nanoparticles), Eosin powder and PBS (phosphate buffered saline) were purchased from Sigma-Aldrich, St Louis, MO, USA and were stored at room temperature until testing. CASPASE 3 activity ELISA kit was purchased from Zellbio, Germany. Albendazole was obtained from Kimiafaam, Tehran, Iran.

### Collection and Extraction of Sea Cucumber

Sea cucumbers (Genus: *H. leucospilota*) were collected in 2016 from the Persian Gulf, around the coastal cities of Kish Island and Bandar Lengeh, Iran. Samples were transferred to Parasitology Laboratory of Urmia University of Medical Science. All of samples were washed and cleaned several times with distilled water. The different parts of *H. leucospilota* (cuvierian organs and body wall) were divided into small pieces. Samples were kept at room temperature a way from light and heat for 2–3 d until completely dry. After that, the different dried parts of *H. leucospilota* were powders by a homogenizer. Then all powders, separately, were put in methanol-water (50:50) for 48 h. The blends were filtered by Buchner funnel and then concentrated by rotary evaporation. Finally, all extracts were dried by a freeze dryer and kept them at refrigerator temperature until testing.

### Collection of cysts

Hydatid cysts of *E. granulosus* were collected from infected livers and lungs of sheep and cattle slaughtered at Urmia abattoir between years 2016–2017 and transferred to Parasitology Laboratory of Urmia University of Medical Science. Cysts were washed and cleaned with distilled water and external part of cysts was sterilized 70% ethanol. The hydatid fluid aspirated from the fertile cysts with a sterile syringe, transferred into tubes, and waited 30 min to settle down protoscoleces. The supernatant phase was removed and the protoscoleces were washed 3 times with sterile phosphate buffered saline (PBS), pH 7.2 (27, 28).

### In-vitro scolicidal effects of CeO2 NPs and *H. leucospilota*

**Fig. 1:**
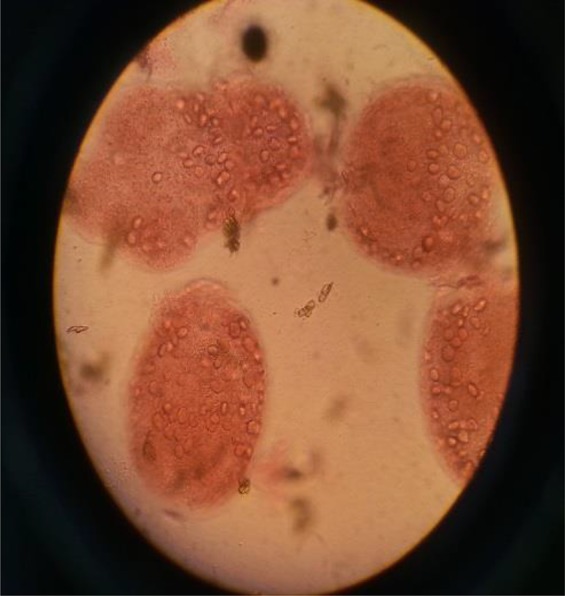
Dead protoscoleces of *E. granulosus* after exposure with 0.1% eosin

**Fig. 2:**
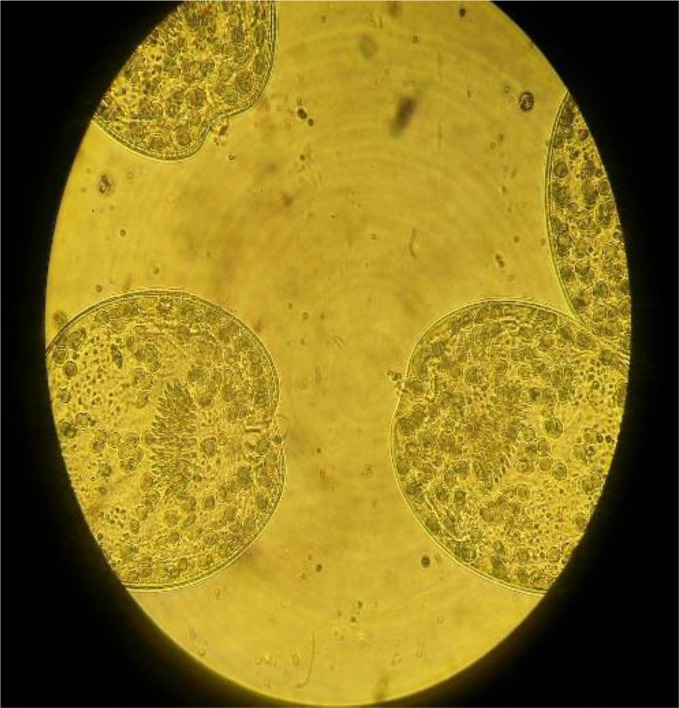
Live protoscoleces of *E. granulosus* after exposure with 0.1% eosin

In present study to evaluate the viability of protoscoleces, 0.1% eosin staining was used. 1ml of 0.1% eosin was added to 1 mL of settled protoscoleces and mixed gently after a few minutes examined microscopically. Alive protoscoleces remained colorless and showed flame cells movements and dead protoscoleces colored red and become shriveled (Figs. 1, 2). In this investigation, four concentration of *H. leucospilota* extract, (1,5,10,20)mg/mL and four concentration of CeO2-NPs (1,5,10,15)mg/mL and four concentration of combination of CeO2 NPs*/H. leucospilota* (1,5,10,15)mg/mL were used for 10,20,30 and 60 min. 0.2 mL of various concentrations of extract and NPs were exposed with protoscoleces in four times (All samples had viability >90% at the time of experiments), and in every experiment, the mortality of protoscoleces was determined (29).

We also used normal saline as a negative control group and albendazole as a positive control group. All experiments were carried out in triplicate.

### CASPASE 3 activity assay detections

Each concentration of *H. leucospilota* extract and CeO2 NPs, were more effective against protoscoleces, were used in CASPASE 3 activity ELISA test. Caspase-3 activity assay detections were performed according to the caspase-3 activity assay kit.

### In-vivo experiments

Male BALB/c mice with weight from 25–30 gr and 8 week old, purchased from Razi institute Tehran, Iran. These mice were kept under normal conditions with a 12:12 h dark/light cycle with access to food and water. Mice were infected intraperitoneally with 2000 viable protoscoleces in /0.5 mL sterile PBS (29, 30). After two months of infection, 5 mice were selected randomly and anesthetized with ether and cysts were seen in peritoneal cavity.

Histopathological analysis was used to prove the layers of hydatid cyst. For this examination, small part of peritoneal cavity cyst fixed in 10% formalin and then stained with hematoxylin-eosin.

The study was conducted in accordance with the ethical standards of “Principles of Laboratory Animal Care with approval cod: Ir.UMSU.REC.1395.153.

### Experimental treatment of *E. granulosus*-infected mice

Infected mice have divided into 5 groups of 10 mice was in each treatment group. Drug concentration were prepared (CeO2 NPs:50 mg/kg, *H. leucospilota*: 200mg/kg, Combination of CeO2 NPs/*H. leucospilota*: 300 mg/kg). Infected mice treated daily for 4 week by intragastrical inoculation (100 Microliter/day). Albendazole with concentration of 50mg/kg as control group. After 4 week, the mice were dissected and evaluated based on the presence or absence of cysts and morphological changes between groups.

### Statistics

All the In-vitro and in-vivo experiments were performed in triplicate. Data analysis were carried out by using GraphPad Prism 7 and SPSS 16.0 (Chicago, IL, USA). The differences between groups were determined using t-test and ANOVA testing and *P*-values less than 0.05 were considered significant.

## Results

### Determination of in vitro scolicidal effects of CeO2 NPs and *H. leucospilota* extract and combination of both by 0.1% eosin stain

**Fig. 3:**
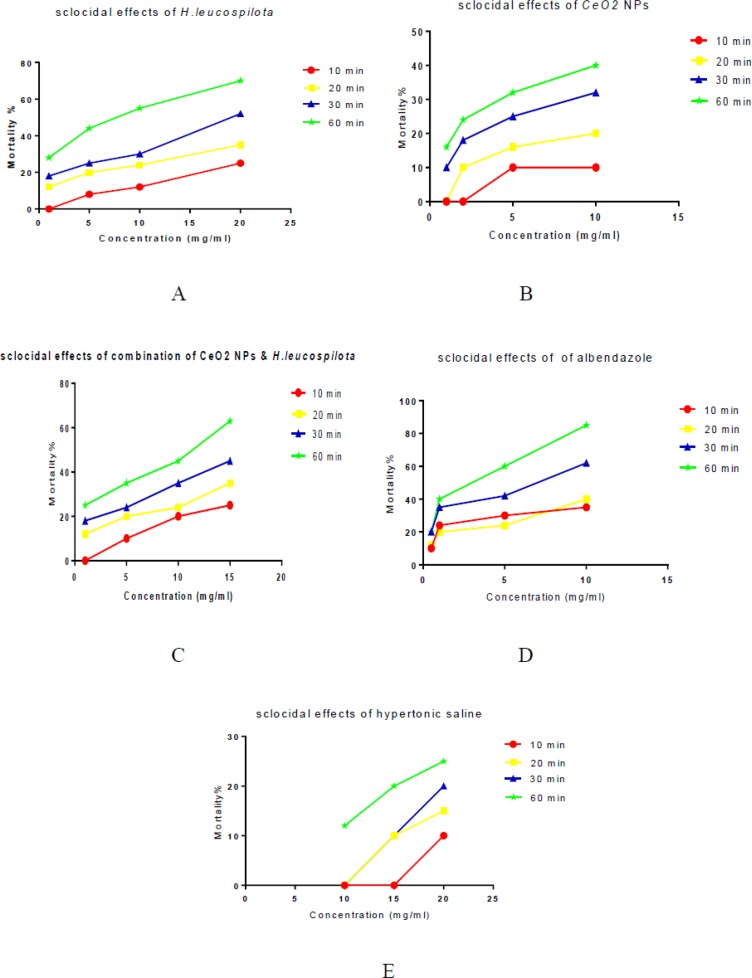
Scolicidal effects of CeO2 NPs and *H. leucospilota* extract in different concentrations and different times **A:** CeO2; **B:** *H. leucospilota* extract; **C:** *H. leucospilota* & CeO2 NPs; **D:** Albendazole, (positive control); **E:** PBS (negative control)

According to Table 1, The *H. leucospilota* extract and CeO2 NPs and combination of *H. leucospilota &* CeO2 NPs were shown potent scolicidal activity against protoscoleces of hydatid cysts under in-vitro conditions with reference to the known standard drug Albendazole in a dose-dependent and time-dependent. Figure 3 shows scolicidal effects of different concentration of the *H. leucospilota* extract (1,5,10,20) mg/mL and CeO2 NPs (1,5,10,15)mg/mL and combination of both (1,5,10,15) mg/mL after 10,20,30 and 60 min against protoscoleces of *hydatid cyst*.

**Table 1:** Mortality rate of protoscoleces (%)

***Time (mg/mL)***	***Concentration***	***10 min***	***20 min***	***30 min***	***60 min***
		***Mortality rate of protoscoleces %***			
*H. leucospilota* extract	1	0	12	18	28
	5	8	20	25	44
	10	12	24	30	55
	20	25	35	52	70
	25	33	44	60	79
CeO2-NPs	1	0	0	10	16
	2	0	10	18	24
	5	10	16	25	32
	10	10	20	32	40
	20	5	8	8	5
CeO2 & *H. Leucospilota* extract	1	0	12	18	25
	5	10	20	24	35
	10	20	24	35	45
	15	25	35	45	63
Albendazole	0.5	10	12	20	20
	1	24	20	35	40
	5	30	24	42	60
	10	35	40	62	85
Hypertonic saline	10	0	0	0	0
	15	0	10	10	10
	20	10	15	20	25

The maximum scolicidal effects of CeO2 NPs and *H. Leucospilota* extract and combination of both were observed at the concentration of 10 mg/mL and 20 mg/mL and 15 mg/mL.

The mortality rate at the negative control group (hypertonic saline) after 60 min of exposure was 25% and the mortality rate at the positive control group (albendazole) at the concentration of 10 mg/mL after 60 min was 85%. All sea cucumber extracts body wall and cuvierian organs have potential antihydatid activity.

### Determination of in vitro effects by CASPASE-3 activity assay

**Fig. 4:**
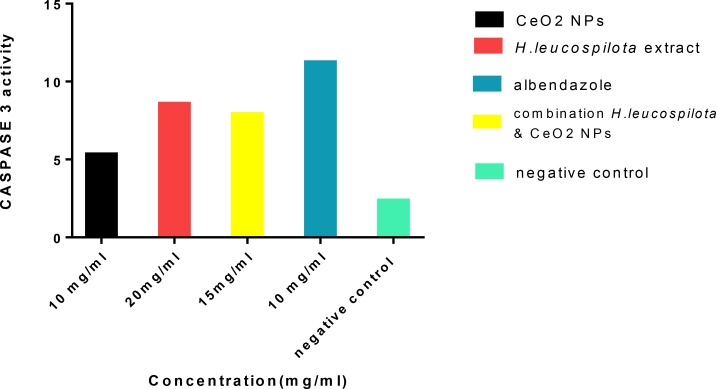
CASPASE -3 activity assay in the three induced groups (*CeO2* NPs: 10mg/ml, *H. leucospilota* extract: 20 mg/ml, combination CeO2 and *H. leucospilota* extract: 15 mg/ml) and control groups (albendazole as positive control group and PBS as negative control group)

CASPASE-3 activity assay was done in the three induced groups and control groups. The concentrations of 10 mg/mL CeO2 NPs, 20 mg/mL *H. leucospilota* extract and 15 mg/mL combination of *H. leucospilota* extract & CeO2-NPs were exposure with protoscoleces for 60 min. After exposure time, Caspase-3 activity assay detections were performed according to the caspase-3 activity assay kit and the apoptotic cells were observed at 450nm with ELISA reader. High enzymatic Caspase-3 Activity correlates with the frequency of apoptotic cells. The number of apoptotic protoscoleces significantly correlated with caspase-3 activity. High caspase-3 activity, as measured using the enzymatic method, was observed when protoscoleces expose with *H. leucospilota* extract and combination *H. leucospilota* extract *&* CeO2 NPs. The minimum level of caspase-3 activity is when protoscoleces expose with CeO2 NPs and Albendazole (Fig. 4).

### In-vivo effects of CeO2 NPs and *H. leucospilota* extract and combination of both on clinical parameters of secondary hydatidosis

**Fig. 5:**
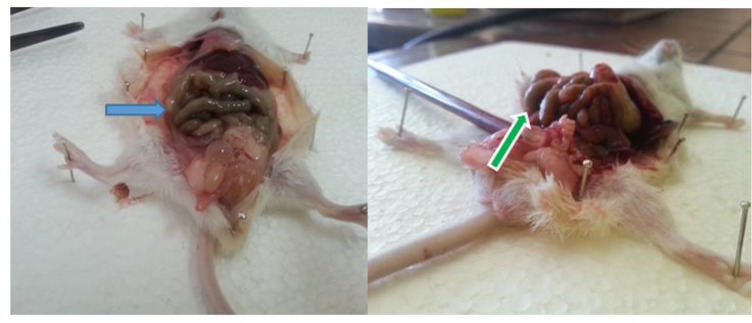
After two months, mice infected with cysts in peritoneal cavity and liver (by injection of 2000 viable protoscoleces in /0.5mL sterile PBS)

After 4 week of treatment period, the mice were euthanized and the peritoneal cavity was opened (Fig. 5), and the hydatid cysts were carefully removed. Size, number and weight of hydatid cysts in different treatment groups were calculated and compared with positive control group (albendazole) and negative control group. According to Table 2, the maximum size, number and weight of hydatid cysts were observed in the group of mice-infected with no treatment period, and the minimum size and number of hydatid cysts and volume range was observed in the group of mice-infected with albendazole and *H. leucospilota* extract and Combination of *H. leucospilota* extract & CeO2 NPs treatment.

**Table 2:** Number, size and weight of hydatid cysts in the different groups

***Experimental groups***	***Number of cysts***	***Size range (mm)***	***Mean size***	***Weight of cysts (mg)***	***Mean weight***	***Percentage of infected mice (%)***	***Localization of cysts***
Control −	0	0	0	0	0	0	0
Control +	17	5–10.5	1.39±7.46	3.8–5.2	0.49±4.43	100(10/10) mice	Liver and peritoneum cysts
Albendazole	10	2.7–6.8	0.90± 3.27	2.8–4.2	0.51±3.41	100(10/10) mice	Liver and peritoneum cysts
CeO2-NPs	8	2.3–5.5	1.14±4.87	3.5–5	0.53±4.25	100(10/10) mice	Liver and peritoneum cysts
*H. leucospilota* extract	6	2.3–5.2	0.98±3.63	2.7–4.4	0.54±3.54	100(10/10) mice	Liver and peritoneum cysts
Combination of *H. leucospilota* extract & CeO2-NPs	8	2.8–5.2	0.98 3.96	3–4.6	0.56±3.65	100(10/10) mice	Liver and peritoneum cysts

## Discussion

Cystic echinococcosis caused by the larval stage of the cestode *E. granolosus*. Now, surgery is the first choice and main treatment for treatment of hydatid cyst however there is increasing risks of frequent recurrences, therefore, use of suitable scolicidal agents with low side effects can be more effective for surgeons (2, 30,31). Antiparasitic drugs treatments such as albendazole and mebendazole are toxic in many cases, study based on the use of some scolicidal agents with no toxic, side effects and its protective abilities. This study for the first time has investigated the scolicidal effects of *H. leucospilota* extract and CeO2 nanoparticles against protoscolices of hydatid cysts on in vitro and in vivo model.

Sea cucumber *H. leucospilota* isolated from Persian Gulf showed pharmacological activities such as antifungal, antivirus, anticancer, antitumor, antioxidant, anti-inflammatory and antibacterial. Thus, sea cucumber with anticancer and anti-tumor activities might be also suitable as antihelminthics agents that can induce apoptotic cell death suggested that can anti-helminthic and anti-hydatid activities (22, 31, 32). In addition, anti-parasitic effects of *H. leucospilota* have already been studied. Methanolic extract of *H. leucospilota* possesses lethal effects on *L. infantum* promastigotes and leads to the inducing of the apoptosis in parasites, *L. infantum* promastigotes were sensitive to *H. leucospilota* extract at different concentrations in time and in dose-dependent manner. Apoptosis occurs in *Leishmania* promastigotes after exposure with *H. leucospilota* extract (22) Our finding showed that *H. leucospilota* extract has a potent scolicidal in vitro and in vivo model (200 mg/kg in vivo and 20 mg/ml in vitro).

In vitro examination showed that effective scolicidal activity of *H. leucospilota* extract and combination of *H. leucospilota* extract at concentration 20mg/ml and 15 mg/ml. Finding demonstrated the in vivo treatment of infected mice with combination of *H. leucospilota* extract /CeO2 NPs and *H. leucospilota* extract had a reduction in the parasite number, size and weight in compared to the control groups with respective *P*-value<0.05 showed that the reduction was statistically significant (Table 2).

Similar to these results, many other new compounds and plants and nanoparticles are known to have activity against hydatid cyst, for example, Garlic *(Allium sativum)* (33), *Artemisinin* (34), *Mentha* spp. essential oil (35), Ajowan (*Trachyspermum*) (36), *Punica granatum* peel aqueous (30), *Berberis vulgaris* root (37), *Mallotus philippinensis* (38),, *Foeniculum vulgare* Mill (27), selenium NPs (39), solid lipid NPs (40) and gold-NPs (41). *Echinacea purpurea, Sambucuebulus* and zinc oxide nanoparticles resulted in a significant decrease in the number of the developed cysts (42). *F. vulgare* essential oil had remarkable scolicidal effects in comparison with positive control; so that, at concentrations of 1 and 0.5 mg/ml killed 100% protoscoleces after 5 and 10 min, respectively (27).

## Conclusion

We observed in vitro and in vivo scolicidal activity of *H. leucospilota* extract and CeO2 NPs for the first time in compared with the positive control albendazole, our extract has potent scolicidal effects against protoscoleces of *E. granulosus* in vitro and in vivo model, but these results should be examined by extent molecular and experimental studies before introducing them as a suitable drug in treating human and animal hydatidosis.
